# Deploying and scaling distributed parallel deep neural networks on the Tianhe-3 prototype system

**DOI:** 10.1038/s41598-021-98794-z

**Published:** 2021-10-12

**Authors:** Jia Wei, Xingjun Zhang, Zeyu Ji, Jingbo Li, Zheng Wei

**Affiliations:** Xi’an Jiaotong University, Xi’an, 710049 Shaanxi China

**Keywords:** Computer science, Computational science

## Abstract

Due to the increase in computing power, it is possible to improve the feature extraction and data fitting capabilities of DNN networks by increasing their depth and model complexity. However, the big data and complex models greatly increase the training overhead of DNN, so accelerating their training process becomes a key task. The Tianhe-3 peak speed is designed to target E-class, and the huge computing power provides a potential opportunity for DNN training. We implement and extend LeNet, AlexNet, VGG, and ResNet model training for a single MT-2000+ and FT-2000+ compute nodes, as well as extended multi-node clusters, and propose an improved gradient synchronization process for Dynamic Allreduce communication optimization strategy for the gradient synchronization process base on the ARM architecture features of the Tianhe-3 prototype, providing experimental data and theoretical basis for further enhancing and improving the performance of the Tianhe-3 prototype in large-scale distributed training of neural networks.

## Introduction

Deep Neural Network (DNN) is the foundation of modern Artificial Intelligence (AI) applications^1^. In recent years, due to the landmark performance of DNNs in natural language processing^2^ and image recognition^3^, they have been widely used in areas such as unmanned vehicles^4^, cancer detection^5^, and complex decision-making^6^ especially in the image domain, where the deep learning-based AlexNet model improves the classification accuracy by a factor of two compared to the traditional algorithm represented by support vector product, thus attracting interest from the image recognition community as well as academia. The superior performance of DNNs stems from their ability to extract high-level features from raw data by performing statistical learning on large amounts of data to obtain an efficient representation of the input space^7–9^. This is quite different from earlier machine learning approaches that used specific features or rules designed by experts. However, the outstanding performance of DNN comes at the cost of high computational complexity. With the increase of the size of the dataset and the improvement of the complexity of the model, the demand for computational strength and storage space of DNN in the training process also increases proportionally^10–12^. Although general computing engine (especially GPU) has become the main means to accelerate DNN training^13–16^, people have a deeper interest in professional DNN training acceleration technology. In order to make the trained DNN more competitive, High-Performance Computing(HPC) clusters are essential^17–19^. For the above system, DNN training and reasoning (evaluation) and other aspects need to be optimized^20–24^ to adapt to the characteristics of the corresponding platform, so as to improve the overall concurrency.

In recent years, high energy-efficient heterogeneous computing platforms^8, 10–25^, represented by GPU, MIC and FPGA, have been widely used in different research fields. The concept of heterogeneous computing is also used to manufacture supercomputers^15^. For example, Tianhe-1 (using GPUs as accelerators) developed by China ranks first in the TOP500 list in November 2010, and Tianhe-2 (using MIC as coprocessor) ranks first in the TOP500 list in June 2013. Shenwei Taihu light supercomputer, which is made of domestic heterogeneous multi-core CPU ranked first in the TOP500 list in June 2016. In June 2020, the Fugaku supercomputer, based on ARM architecture and equipped with CPU “A64FX” developed by Fujitsu, won first place in the latest list of t500 in 2020 with a computing speed of 415.5 petaflops. So far, the development of supercomputing to E-level is still a great challenge for the whole high-performance computing (HPC) community. Whether it can show good applicability to the deep neural network is also the focus of many research institutions and industries.

In the Chinese E-scale plan, Tianhe-3 uses an ARM-based multi-core architecture, and the processor uses domestic Phytium-2000 + (FTP) and Matrix-2000 + (MTP) and is open to the public for performance evaluation.

The rapid development of HPC provides a platform foundation for the parallelization of deep neural networks, and a rich parallel programming framework bridges its parallelization. Therefore, how to combine the algorithm characteristics of deep neural networks and the architectural characteristics of HPC clusters? It is very urgent to use a parallel programming framework to design neural network distributed computing methods that can give full play to the computing power of high-performance platforms. In order to achieve an optimized design to give full play to the high performance of the supercomputing platform, it is first necessary to perform corresponding evaluation and tuning on the specific high-performance computing cluster. This paper evaluates and optimizes the distributed training performance of neural networks on the Tianhe-3 Prototype platform.

The contribution of this paper is as follows:

- Designed a series of targeted experiments by considering three dimensions: neural network model, neural network execution framework, and test pattern, in response to the unique architectural characteristics of the Tianhe-3 prototype.
- The distributed framework of Pytorch was ported to the Tianhe-3 prototype and adapted to the Tianhe MPI.
- Different test strategies were designed and implemented for various cases such as single MTP node, single FTP node, multiple MTP nodes, and multiple FTP nodes, respectively.
- A Dynamic Allreduce Algorithm communication optimization strategy is proposed for the gradient synchronization process of distributed data parallel deep learning.

This paper is organized as follows: “Related work” section describes about distributed parallel deep learning training in single and multi-node, “Key issues” section introduces mini-batch gradient descent, parallel model in distributed environment, consistency computing model and the basic information of the Tianhe-3 prototype system, “Dynamic Allreduce algorithm” section introduces our proposed Dynamic Allreduce Algorithm, “Experimental design” section presents our experimental design in terms of neural network model, neural network execution framework and test model, “Experimental evaluation” section shows our experimental results in the Tianhe-3 prototype using the experimental design in “Experimental design” section for single and multiple nodes, and provides a detailed analysis of the extensibility, computation and communication overhead of the algorithm, and finally, the full paper is summarised in “Conclusion” section.

## Related work

With the increasing task scale and the deepening of network layers, the distributed training of the deep neural network has experienced the development from a single node to multi node. On a single node, multi-core and multi-core technology (GPU, MIC) is used for parallel acceleration; for large-scale deep neural network training tasks that cannot be completed by a single node, MPI or spark^9^ is usually used to complete the training of the whole model through the distributed system. In the parallel acceleration training process, people focus on using ASIC, FPGA, or cluster^17, 25–28^.

In terms of GPU acceleration, Raina et al.^15^ first proposed to use GPU to accelerate deep Boltzmann machine (DBN); Yadan^16^ and others used four NVIDIA Geforce GTX Titan GPUs on a single server to realize the combination of data parallelism and model parallelism; Li^14^ and others used a single Tesla k40c GPU to accelerate DBN, and compared with Intel Core i7-4790k, it obtained 14–22× speedup the ratio in the pre-training process; Bottleson et al.^13^ proposed an OpenCL accelerated Caffe framework for CNN network, and compared with Intel CPU, it obtained 2.5–4.6× speedup after different optimization methods.

In terms of MIC acceleration, OpenMP parallel programming model is generally used. Olas et al.^29^ ran DBN in native mode on Intel Xeon phi 7120p, and achieved 13.6× speedup compared with the serial version of Intel Xeon e5-2695v2 CPU under 240 threads. Zlateski et al.^30^ proposed a 3D convolution algorithm, which achieved near-linear speedup on Intel CPU and 90× speedup on 5110P. In terms of FPGA acceleration, Suda et al.^27^ has implemented two kinds of networks, VGG and Alexnet on FPGA, among which VGG is 5.5× compared with Intel i5-4590 3.3 GHz CPU. Zhang et al.^28^ implemented VGG network on FPGA, and the computing performance of VGG network on Altera Arya 10 reached 1.79tops, and obtained 4.4× acceleration ratio compared with Intel Xeon e5-1630v3 CPU. Utkuaydonat et al.^26^ implemented Alexnet network on FPGA, optimized the out of chip memory bandwidth, and introduced the Winograd conversion method, and finally reached the peak performance of 1382 GFLOPS when running on ARRIA 10.

In terms of Multi Nodes parallelization acceleration, deep neural network multi node parallelization is mainly on the CPU cluster. The classic work includes Google’s dean et al. Who developed Google’s deep learning software framework distbelief on the CPU cluster. Kaida song et al. Trained the DBN network with data parallel strategy on the domestic supercomputer Taihu Light, and trained the MNIST data set on four computing nodes. The performance achieved is 23 times higher than that of Intel Xeon e5-2420v22.2g CPU^26^. Ammar et al.designed a distributed deep learning framework S-caffe with 12 nodes and 80 Tesla K80 GPUs using the method of collaborative design^25^. Fang Jiarui et al. Proposed Swcaffe, transformed and transplanted the Caffe framework to Taihu Light, and obtained processing speed exceeding nvk40m GPU in some neural network structures^17^. Moritz et al. Realized the combination of spark and Caffe, and improved the traditional synchronous SGD parallelization mechanism by introducing control parameters to reduce the synchronization times^18^.

Testing and tuning supercomputers have always been an important and challenging topic in the HPC field. Peng et al.^19^ implemented Amber parallel acceleration strategy for the molecular dynamics model on Tianhe-2 computer, realizing 25–33 times acceleration of the original program; Yifeng Xu et al.tested ZnO MOCVD cavity numerical model on Tianhe-2 platform and the maximum acceleration ratio was 45. Zhu et al.^24^ studied the distributed extension of Caffe, a deep learning framework, for the supercomputing platform of “Shenwei Taihu Light”. They compared the distributed expansion method of parameter server and decentralized distributed expansion method under synchronous mode. Their experiments show that the decentralized distributed extension method is more effective than the distributed extension method of parameter server in synchronous mode. It has obvious advantages in the aspect of signal efficiency, and the communication performance can be improved by 98 times for a specific model. you et al.^23^ tested and analyzed the computing performance of linear algebraic kernel for Tianhe-3 Prototype, and compared the performance of MTP and FTP on Tianhe-3 Prototype and KNL processor for comparison. Li et al.^22^ proposed a heuristic topology-aware mapping algorithm Kohtma to evaluate and improve the communication performance of Tianhe-3 Prototype. Their experiments show that this method can greatly reduce the communication cost.

## Key issues

In this section, we mainly introduce four key points of distributed training in this paper: small-batch gradient descent, parallel model in a distributed environment, consistency computing model and Tianhe-3 Prototype.

### Mini-batch gradient descent

In the process of deep neural network training, a very important task is to solve the cost function (that is, to solve the weight of each layer of the network). At present, the most commonly used optimization algorithm is a gradient descent algorithm. The core of the algorithm is to minimize the objective function by iterative convergence. In each iteration, the corresponding parameter value of each variable is updated according to the objective function in the opposite direction of the variable gradient. Among them, the parameter learning rate and the target optimization index determine the number of iterations to reach the minimum value. There are three different variants of the gradient descent method: Batch Gradient Descent (BGD), Stochastic Gradient Descent (SGD), and Mini Batch Gradient Descent (MBGD, as shown in Algorithm 1). BGD can ensure that it converges to the global optimal value of a convex function or local optimal value of a nonconvex function, but each update needs to be solved on the whole data set, so the speed is slow. Even for large data sets that cannot be stored in memory, this method can not be used, and the model can not be updated in the form of a line. Each update of SGD only solves the gradient of one sample in the data set and runs Compared with BGD, SGD is easy to fall into local minima, and the convergence process is more fluctuating. MBGD combines the advantages of the above two methods. In each update, MBGD solves a batch of data composed of n samples to make the convergence process more stable. It is usually the preferred algorithm for training neural networks. Therefore, in this paper, MBGD is chosen as the method to solve the cost function.

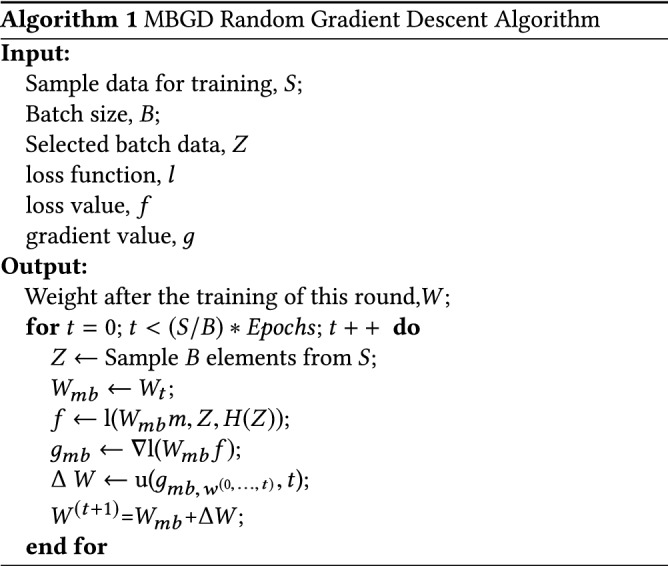



### Parallel model in distributed environment

**Figure 1:**
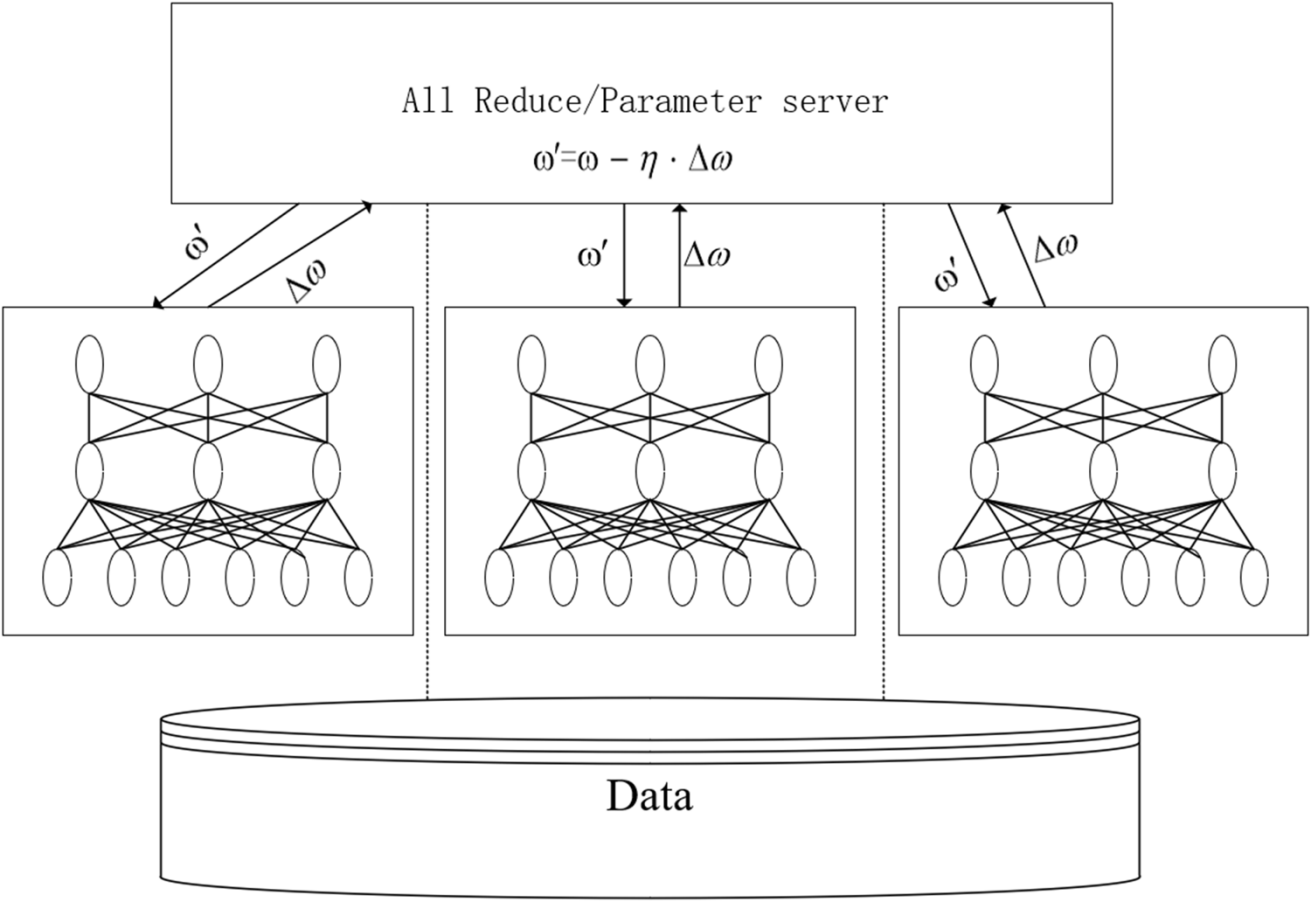
Deep neural network data parallel.

There are two main methods for the parallel of deep neural network: model parallel and data parallel^20^. Model parallelism refers to the decomposition of network model to each computing device, and the training is performed by the cooperation between devices. Model parallelism is more suitable for large models with many levels, few dependencies between levels, and where a single compute node cannot complete the entire network training. Data parallelism refers to the segmentation of training data, and the parallel training of multi segmented data is conducted by using multiple model examples. As shown in Fig. 1, in the data parallel training process, each node uses its own data to train the entire network for one epoch, then perform gradient averaging and update the local weights and move on to the next epoch. $\omega$ represents the wieght value and $\eta$ represents the learning rate. The parameter exchange is completed by parameter server or by using decentralized communication mechanisms such as all-reduce. In MSGD, the data is processed in n samples as batches. Since most operators are independent from n, the direct way to parallelize is to allocate small batches of samples between multiple computing resources (processor cores or devices) to perform work. This method (initially called pattern parallelism, and input samples as patterns) can be traced back to the first practical implementation of artificial neural networks. It can be considered that the use of micro-batch in gradient descent is initially driven by data parallelism. Farber and Asanović^31^ use multiple vector accelerator microprocessors (Spert II) to perform error reverse propagation in parallel to speed up neural network training. In order to support data parallelism, they proposed a delayed gradient update version called “bundle mode”, in which the gradient was updated several times before updating the weight, essentially equivalent to MSGD.

Raina et al. First mapped DNN computing to data parallel architecture (such as GPU). They focus on training deep belief networks and map the unsupervised training process to GPU by running MSGD. Their training results on the restricted Boltzmann machine increased the CPU speed by 72.6 times. Nowadays, most deep learning frameworks support deep data parallelization. They can use a single GPU (CPU) or a cluster of multiple GPU (CPUs) nodes. With MapReduce, parallel tasks can be scheduled to multiple processors and distributed environments. Prior to these works, researchers have studied the potential scale of MapReduce for various machine learning problems, including neural networks, which promotes the need to shift from single-processor learning to distributed storage systems.

For the Pytorch training platform used in this article, it supports data parallelism, training a copy of the model independently on each device. When updating the parameters, the gradient of each device is transmitted to the parameter device for parameter updating. The 1bit SGD algorithm is supported to reduce the amount of data transmitted.

### Consistency computing model

With the increasing amount of data, a single machine can not store all the data, so a distributed optimization algorithm has become a hot spot. Researchers have proposed parallel optimization algorithms based on parallel computing models such as bulk synchronous parallel (BSP)^32^, delay synchronization (SSP)^33^, and asynchronous (ASP)^33^.

The global synchronous parallel computing model BSP is composed of a group of distributed processors with local memory and roadblocks supporting global synchronization between all processing units. The synchronous update process is as follows: multiple processor units update the model in the same iteration round. After completing a round of iteration, the multiprocessor units wait synchronously at the master node according to the roadblock mechanism. The master node updates the model uniformly and sends the updated parameters to each processor unit for a new round of iteration. Common BSP model systems include mahout^34^, spark^34^, etc.

The asynchronous parallel computing model ASP is composed of a distributed processor with local memory and a total node managing global parameters. The asynchronous update process is: each node updates the model on the master node in an asynchronous manner. Combined with the data division, it can be explained that: each node calculates all model parameters with local data. After one round of calculation, the node can update the model parameters at the master node, and then get the latest global parameters from the master node for the next round of updating. Each node does not need to wait for each other. However, there is no limit on the number of iterations of each node, which will result in the final non-convergence. In order to solve the instability of the ASP model, the following delay synchronization parallel computing model is proposed.

Delay synchronous parallel computing model SSP is composed of a distributed processor with local memory, total node and delay parameter (stale). The semi asynchronous update the process is: each node updates the model on the master node with different rounds, but with a little restriction, the iteration rounds of the parameters of the fastest and slowest nodes should not be greater than stale, so that the slow nodes can not only slow down the running speed of the whole system but also ensure the final convergence of the model parameters. Common SSP model systems include Petuum^35^, deep learning system^20, 36, 37^, etc.

**Figure 2:**
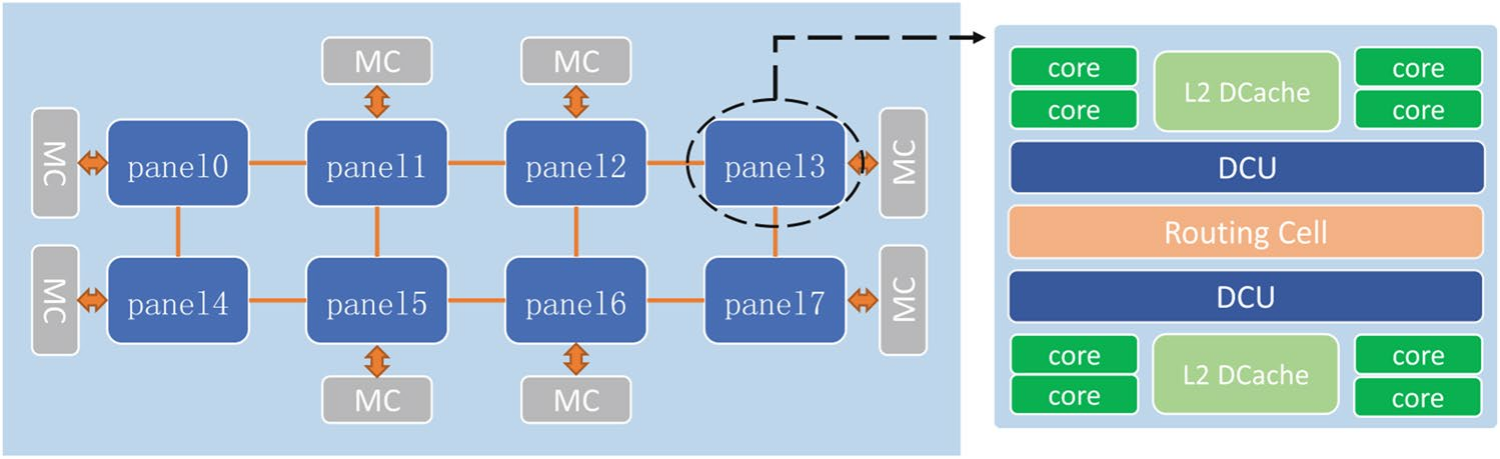
FT2000+ processor architecture.

**Figure 3:**
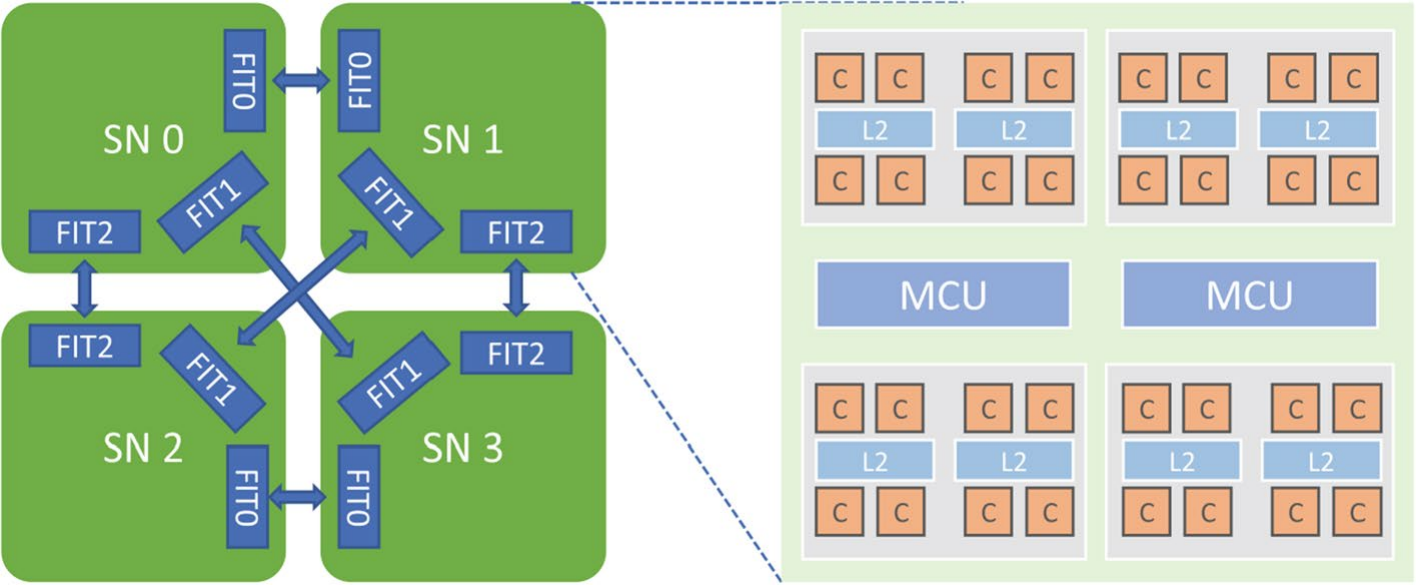
MT2000+ processor architecture.

### Tianhe-3 prototype

**Table 1:** Basic situation of Tianhe-3 prototype system.

Specifications		FT-2000+	MT-2000+
Hardware	Nodes	128	512
	Cores in a node	32	32
	Frequency	2.4 GHZ	2.0 GHZ
	Memory	64 GB	16 GB
	Interconnect bandwidth	200 Gbps	200 Gbps
Software	OS	Kylin 4.0-1a OS with kernel v4.4.0	
	File system	Lustre	
	MPI	MPICH v3.2.1	
	Compiler	GCC v4.9.1/v4.9.3	
	Supported libraries	Boost, BLAS, OpenBLAS, Scalapack, etc.	

As shown in Figs. 2 and 3, the processors used in the Tianhe-3 Prototype include FT-2000+ (FTP) and MT-2000+ (MTP). In the official website of Tianjin Supercomputing Center and Feiteng Company, as well as published papers, you can know that FTP contains 64 FTC662 processor cores with armv8 architecture, the main frequency is 2.2-2.4GHZ, the on-chip 32MB secondary cache is integrated, which can provide 204.8GB/s memory access bandwidth and the typical working energy consumption is about 100W; while the MTP processor, It contains a total of 128 customized processor cores, organized into 4 super nodes, the main frequency is up to 2.0GHZ, and the entire processor consumes 240W. In addition, both processors support 128-bit SIMD vector or scalar floating point operations, which is critical for deep learning. The processor architecture of FTP and MTP is shown in the figure below:

In Tianhe-3 Prototype, as shown in Table 1 below, FTP and MTP are both divided into 32 cores as one computing node. The purpose of doing so may be to provide more computing nodes to meet complex computing tasks^23^. The computing nodes are managed and allocated by the batch scheduling system. In FTP, 32 cores share 64g of memory, while in MTP, 32 cores share 16g of memory. They all come with Kylin 4.0-1a operating system with kernel v4.4.0.

In addition, the Prototype cluster interconnection technology designed and implemented by the University of Defense Science and technology provides 200gbps bidirectional interconnection bandwidth. The Prototype uses Lustre for distributed file system management. At the same time, the Prototype provides multiple versions of MPI compiler implementation, including MPICH 3.2.1 (supports gcc4.9.3, gcc8.2.0, and gcc8.3.0) and an openmpi4.0.0 which is still in the debugging stage. In addition, lammps, GROMACS, and other scientific computing applications are preset.

Unfortunately, the Prototype does not provide development frameworks for deep neural networks such as Caffe, Pytorch, Tensorflow, etc. Therefore, the work of this article first starts with the transplantation of the Pytorch framework.

## Dynamic allreduce algorithm

**Figure 4:**
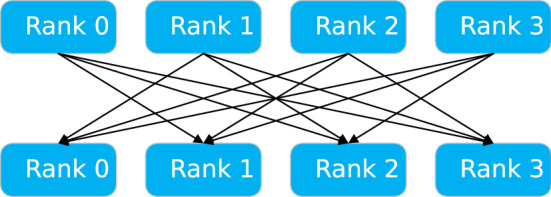
Pytorch distributed Allreduce method.

In the distributed data parallel deep learning training process, each node has to perform gradient averaging after each small batch of data training to obtain the training results of each node to update the network weights, and this process is now often done using parameter servers or Allreduce operations for aggregate communication. The existing Pytorch distributed underlay uses an all-broadcast-like Allreduce strategy as shown in Fig. 4 (We can use it easily by calling the $torch.distributed.all\_reduce()$ API.). Although this strategy can alleviate the problem that parameter server models are prone to single-point-failure communication bottlenecks, this approach increases the amount of communication data by a factor of n-1 (n is the number of nodes), and at larger data blocks (as the neural network model becomes more complex, the model gradient of updated data increases) are prone to delay jitter.

**Figure 5:**
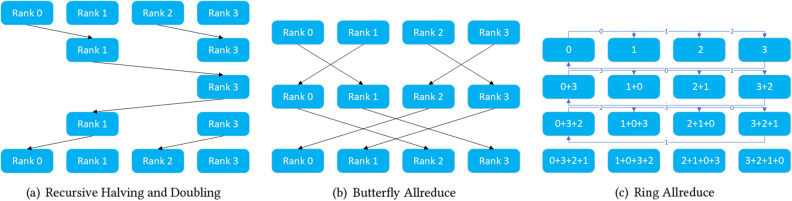
Recursive halving and doubling, butterfly and ring Allreduce.

As a result, we combine the architectural features of Tianhe-3 and introduce the three most commonly used current pooled communication underlying strategies of Recursive Halving and Doubling, Butterfly and Ring AllReduce. As shown in Fig. 5, different strategies show different performance variations when exchanging data size, interconnection method between nodes, sending and receiving buffer size, node processor type, etc. Generally, with the same communication bandwidth, the communication throughput will show a non-linear variation with the size of the communication data block, and the communication speed will be fastest when the size of the model matches the size of the data volume corresponding to the maximum value of the communication throughput. In the past, people normally choose based on experience, but this requires researchers to have sufficient knowledge of the underlying communication mechanisms and implementations as well as the size of the exchanged data, which is often not a necessary skill for deep learning researchers.

In light of this, we take full advantage of distributed training to design and implement a probe mechanism that allows users to automatically select the most suitable underlying communication method without the need to know the underlying implementation of the communication.

As shown in Algorithm 2 below, after all compute nodes have completed data preprocessing, they communicate using the original Allreduce, Recursive Halving and Doubling, Butterfly, Ring AllReduce, and Parameter Server when executing the first 5 batch synchronization gradients, respectively, and the total communication time for each strategy is saved in the local probe. In each subsequent training round, the gradient averaging is done using the communication strategy with the shortest communication time obtained from the above four probes. At the same time, if the number of neurons in each layer of the neural network model varies greatly, resulting in uneven amounts of simultaneous gradient data in each layer, it is also possible to probe deeper into each neural network layer and specify a specific underlying communication strategy for each layer of the neural network gradient averaging operation. Specifically, if the underlying communication policy needs to be optimised layer by layer, we simply create a probe for each layer as described in Algorithm 2. Similar to the optimisation for the whole network, the communication times of the different underlying communication policies are stored in the probes of each layer separately, and then each layer probe selects the policy with the shortest communication time in its layer as the underlying communication policy for gradient synchronisation in the subsequent training process of each layer. 
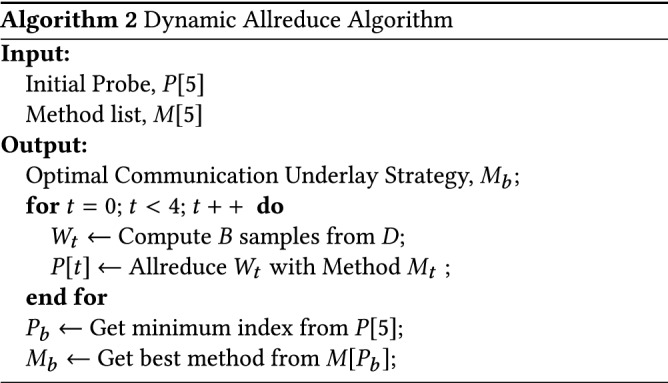

## Experimental design

We propose a targeted experimental design by considering three dimensions: neural network model, neural network execution framework and test pattern to fully test the performance of the Tianhe-3 prototype in distributed training of deep neural networks.

### Neural network model

To fully test the performance of the Tianhe-3 prototype in distributed training of deep neural networks, As shown in Table 2, we first selected a modified version of the LeNet model to test in detail the performance of the Tianhe-3 single-node and multi-node implementations when working on image classification of the Minist dataset, which contains two convolutional layers (conv layers), two pooling layers, three activation layers, and two fully connected layers(fc layers). The first convolutional layer, with an input channel count of 1 and an output channel count of 10, has a convolutional kernel size of 5x5, a stride size of 1, and zero padding. The second convolutional layer has 10 input channels and 20 output channels, and the rest is the same as the first convolutional layer. Both pooling layers use the maximum pooling method. All three activation functions use the relu function. The drop_out optimization method is also used for the model. Subsequently, we also verify the scalability of Tianhe-3 by using the classical AlexNet, VGG16 and ResNet18 models for image recognition on the Cifar-10 dataset. We use a fixed batch size of 128 for mini-batch stochastic gradient descent, and the number of training samples per batch for each training process is 128/n (n represents the number of processes involved in training).

**Table 2:** Neural network configuration.

Model	Conv layers	FC layers	Dataset
LeNet	2	2	Mnist
AlexNet	5	3	Cifar-10
VGG16	13	3	Cifar-10
ResNet18	17	1	Cifar-10

### Neural network execution framework

We first ported the Pytorch^38^ distributed deep neural network training framework to the Tianhe-3 prototype platform. Pytorch has a fairly simple, efficient, and fast framework that is designed to pursue minimal packaging, conform to the human mind, and allow users to focus on implementing their ideas as much as possible. Pytorch provides a rich library of deep learning kernels, which allows us to quickly and easily build deep learning networks and implement training and inference, for example, by calling the Torch.nn API to easily implement Conv, Pooling, Linear, Batchnorm, Dropout and other basic operations commonly used in deep learning. Pytorch supports the use of MPI, GLOO or NCCL (Nvidia GPUs only) underlying communication frameworks, and we used four MTP nodes to train 10 rounds of the task of classifying Cifar-10 images in AlexNet networks using each of the two frameworks, and the results are shown in Table 3 below. The training time using the GLOO framework is more than five times that using the MPI framework, so we chose MPI, which is more suitable for Tianhe-3, as the distributed underlying communication architecture. Meanwhile, this determines that we can only deploy pytorch on Tianhe-3 using the source installation.

**Table 3:** AlexNet using GLOO framework or MPI framework to classify the Cifar-10.

Communication framework	Training time (mins)
MPI	141.55
GLOO	732.94

The datasets we use for training are the Mnist dataset and the Cifar-10 dataset, a very classical dataset in the field of machine learning, consisting of 60,000 training samples and 10,000 test samples, each of which is a 28$\times$28 pixel grayscale handwritten digital image. CIFAR-10 is a small dataset for recognizing pervasive objects compiled by Hinton’s students Alex Krizhevsky and Ilya Sutskever, a small dataset for identifying pervasive objects. A total of 10 categories of RGB color images are included: airplanes, cars, birds, cats, deer, dogs, frogs, horses, boats, and trucks. The size of the images is 32$\times$32, and there are 50,000 training images and 10,000 test images in the dataset.

### Test model

Tianhe-3 Prototype has two different processor nodes: MT-2000+ and FT-2000+. This paper designs MT-2000+ and FT-2000+ single node and multi-process parallel training tasks, MT-2000+ multi-node and multi-process distributed training tasks and FT-2000+ multi-node and multi-process distributed training tasks to a comprehensive evaluation of single-node parallel training performance and the scalability of multi-node distributed training on the Tianhe-3 prototype.

We can get the Scale-up capability of the Tianhe-3 prototype through a single-node multi-process training design, the Scale-out capability through a multi-node single-process training design, and finally, the capability under both Scale-up and Scale-out through a multi-node multi-process training design.

In order to ensure the robustness of the data, all experimental results in this paper are the arithmetic mean after five tests.

## Experimental evaluation

**Figure 6:**
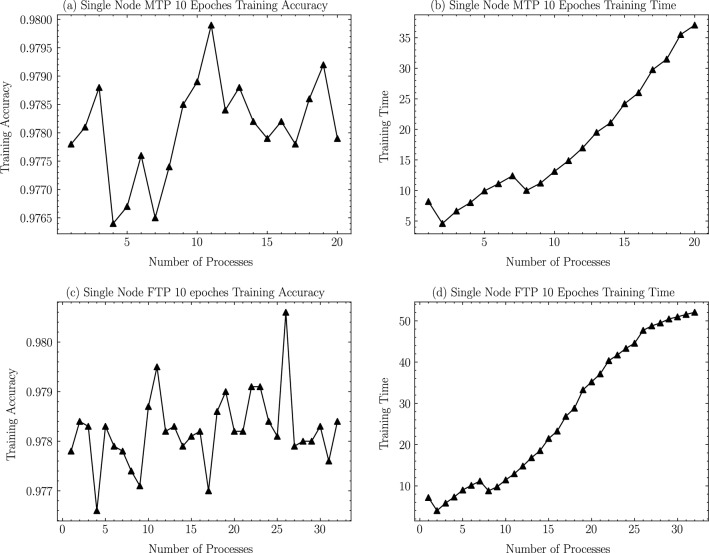
MT2000+ and FT2000+ single node training accuracy and training time.

**Figure 7:**
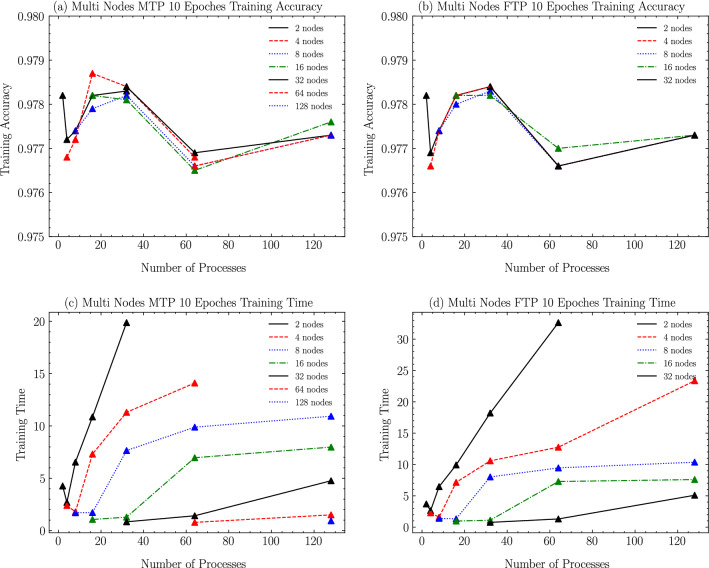
MT2000+ and FT2000+ multi nodes training loss and training time.

**Figure 8:**
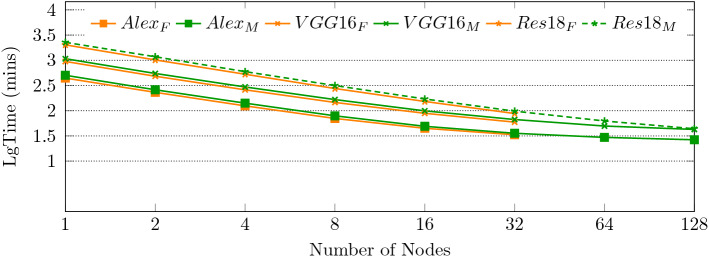
AlexNet, VGG16, and ResNet18 trainig time.

**Figure 9:**
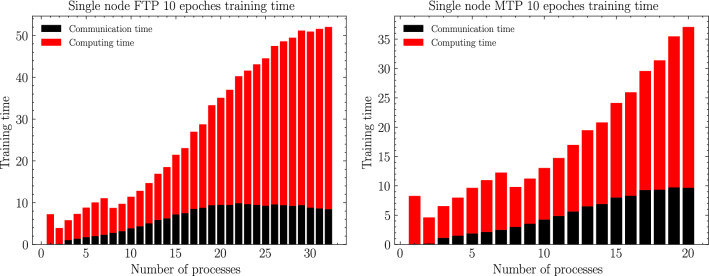
MT2000+ and FT2000+ single node computing and communication time.

**Figure 10:**
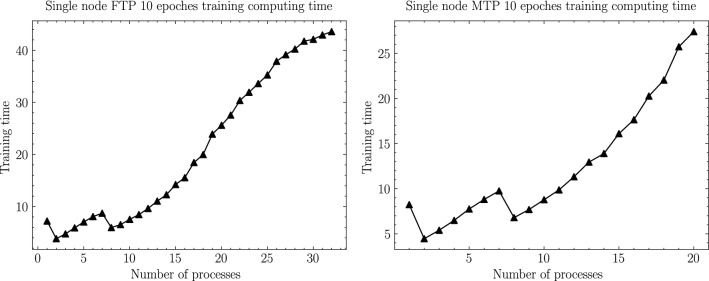
MT2000+ and FT2000+ single node computing time.

**Figure 11:**
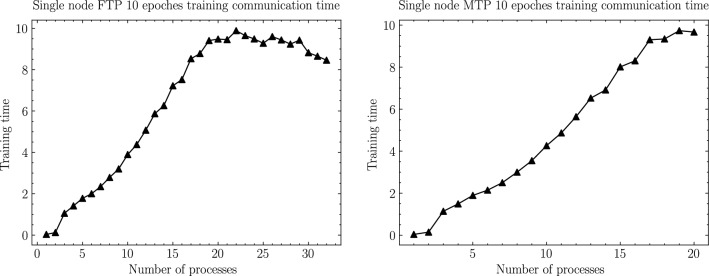
MT2000+ and FT2000+ single node communication time.

In this section, according to the experimental scheme designed in the previous section, as shown from Figs. 6, 7, 8, 9, 10, 11, experiments are carried out for MTP, FTP single node and multi nodes, and the experimental results are summarized and analyzed, and the reasons for the experimental results are explained. Training times for all experiments are counted in minutes.

### Single node performance

In order to fully explore the performance on different multicore processor nodes of MT2000+ and FT2000+, we used a minimum of 1 process to a maximum of 32 processes on one node of two different processors (according to the architectural characteristics of Tianhe-3, a node uses a maximum of 32 processor cores, wherein our experiments MTP can only use a maximum of 20 processes, otherwise it will lead to memory insufficient resources) to classify the Minist dataset using the LeNet model described in the previous section. We adopt a distributed training strategy with data parallelism, an Allreduce communication strategy, and use a strict consistency synchronization protocol (BSP) while shuffling the data on the training set and distributing it evenly over the processes. To ensure consistent training accuracy when testing across multiple processes, we ensure that the same random number is obtained for each experiment by setting a fixed random seed at the beginning of the program.

The accuracy of training in a single MT2000+ node with 10 iterative rounds using 1–20 processes is shown in Fig. 6a. The accuracy results obtained with different numbers of processes are 97.82% on average, and the extreme difference is only 0.04%, which proves the accuracy and robustness of distributed training in a single node of MT2000+. As shown in Fig. 6b, the corresponding total training time is 4.6025 mins when the number of processes is 2, and then the overall training time basically increases with the number of processes (decreases when the number of processes is 8), reaching a maximum of 37.0641 mins when the number of processes is 20; meanwhile, it is found that when the number of processes is a power of 2, the training results are better than the adjacent processes (this is because when The number of nodes that are not a power of 2 when performing parameter synchronization first performs an additional operation to scale the number of nodes to a power of 2 increasing the additional training time).

In a single FT2000+ node, 1–32 processes are used for 10 iterations, and the accuracy of training is shown in Fig. 6c. The average accuracy of different processes is 97.81%, and the range is only 0.035%, which proves the accuracy and robustness of ft2000 + single node distributed training. According to the total training time, as shown in Fig. 6d, the shortest training time is 3.9958 minutes when the number of processes is 2, and then the overall training time increases with the increase of the number of processes (decreases when the number of processes is 8), and reaches the maximum of 52.0821 minutes when the number of processes is 32. Similar to the result in MTP, when the number of processes we use is a power of 2, the training result is obviously better than the selection of adjacent processes.

### Multi nodes performance

As shown in Fig. 7a and c, in the MT2000+ multi-node training process, when the total number of nodes used is less than 8, the shortest training time can be achieved when the number of processes is twice the number of nodes, while Accuracy remains basically the same; when the number of nodes is greater than or equal to 8, the shortest training time can be achieved while minimizing the Accuracy value by choosing the number of processes consistent with the number of nodes.

As shown in Fig. 7b and d, in the FT2000+ multi-node training process, when the total number of nodes used is less than or equal to 8, similar to MTP, when the number of processes is twice the number of nodes, the shortest training time can be achieved while Accuracy remains basically constant, but this time will gradually approach the training time when the number of processes is equal to the number of nodes as the number of nodes increases.

### Extensibility verification

To verify the scalability of the Tianhe-3 supercomputer, we also extended the training scheme in the previous section to typical AlexNet, VGG16 and ResNet18 models, and completed the image recognition task on the Cifar-10 dataset using 1-32 FTP and 1-128 MTP, respectively. The training time for different number of nodes is shown in Fig. 8. The training time decreases with the increase of the number of nodes, showing the good scaling capability of Tianhe-3.

### Overall result analysis

Based on the test results in the previous section, we found that in the performance of single nodes, both FTP and MTP, the results are better when using a power of 2 for the number of processes than the neighboring process number choices (less training time to obtain the same training accuracy). After ten epochs, both processor single nodes achieve optimal training results at a number of processes of 2. At this point, the classification accuracy of FTP is slightly higher than that of MTP by 0.03%, and the single simultaneous training time is reduced by about 13%. When using single nodes, FTP training was faster than MTP regardless of the number of processes used. In the multi-node performance, when the number of nodes used in FTP is less than 16, the results are consistent with those of a single node, and using twice the number of processes as the number of nodes can achieve the shortest training time while the classification accuracy is basically constant. In MTP multi-node training, after the number of nodes is greater than or equal to 8, using the same number of processes as the number of nodes can achieve the shortest training time without losing training accuracy. In addition, we also find that using more nodes, when the number of processes used is the same because more processor cores can be used, must be able to achieve a shorter training time without losing the classification accuracy again.

The advantages and disadvantages of the various architectures are shown in Table 4.

**Table 4:** The advantages and disadvantages of the various architectures.

Distributed training architecture	Advantages	Disadvantages
Single-node multi-process (scale-up)	Simple, cheap	Slow
Multi-node single-process (scale-out)	Simple, medium speed	Expensive
Multi-node multi-process (both)	Fast	Expensive, complicated

The difference in training performance between single-node and multi-node training with different numbers of processes is explained in the next section by analyzing the specific computation time and communication time. A plain reason is that when using single-node training, the computational performance of the nodes is better utilized with two processes than with a single process, but as the number of processes continues to increase, the loss of inter-process communication overhead and the additional overhead caused by inter-process access conflicts outweigh the computational performance gain from increasing the number of processes, resulting in an overall increase in training time; the reason is the same as in single-node when the number of MTP nodes is less than 8 and the number of FTP nodes is less than or equal to 16. When the number of FTP nodes is less than or equal to 16, the reason is the same as in a single node; when the number of nodes continues to increase, even twice the number of processes causes the loss of communication overhead to outweigh the gain in computational performance, so using one process per node is the best choice in this case.

### Computing and communication cost analysis

Further, in order to fully investigate the reasons for the above experimental results, we divided the whole experiment into computation time T1 (which mainly includes the computation overhead of forward and backward propagation of the neural network model and loss value calculation) and communication time T2 (which mainly refers to the communication overhead of global gradient update using Allreduce), and the total time T is the sum of the above two overheads. In FTP and MTP single nodes, the two computational overheads and communication overheads are shown in Fig. 9.

1 $$\begin{aligned} T=T1+T2 \end{aligned}$$

Among them, as shown in Fig. 10, the computational overhead T1 is the overhead that must be paid in order to train the network. As the number of processes increases, the number of training subsets assigned to a single process will decrease, and thus the overhead of forward-propagation and backward-propagation computational operations (including convolution, pooling, activation operations, local gradient update, loss value update, etc.) of the neural network required in a single process will decrease; however, at the same time, as the However, at the same time, as the number of processes increases, the Cache hit rate of each process will significantly decrease and the access conflict will significantly increase according to the shared memory and shared Cache architecture features of the Tianhe-3 prototype. When the number of processes is first incremented, the impact of the former is greater than the latter, so the computation time will increase as the number of processes increases; subsequently, as the number of processes continues to increase, the impact of access conflicts will be much greater than the gain in computation overhead, so the computation time will rise significantly with the increase in the number of processes. Therefore, this leads to the trend that the computation overhead decreases and then increases with the increase of the number of processes.

Similarly, as shown in Fig. 11, the communication overhead T2 is the loss on the path in order to synchronize the weights in the neural network, and it can be calculated using the following equation:

2 $$\begin{aligned} T2={{K}\times \log }_2{{N}\times \left( \alpha +{S}/{B}+S\times C\right) } \end{aligned}$$

Among them, $\alpha$ represents the communication delay between the two processes. It will vary with the distribution and relative distance of the processor cores on the chip. Processor cores sharing the same cache will communicate significantly faster than those sharing only the same shared memory, while those sharing the same shared memory will communicate significantly faster than those using different shared memory but within the same node, and those between two different nodes will communicate the slowest. *S* (ize) represents the size of the data block contained in each process when performing All-reduce operations, *B* (andwidth) represents the communication bandwidth between the two processes, and *C* (omputing) represents the computing overhead per byte (note that this Part of the computing cost refers to the computing cost in the All-reduce process, not the computing cost mentioned in the previous section), *N* (numbers) represents the number of all processes that communicate, and *K* represents the number of All-reduce operation. In our experiment, the Mnist data set used has a total of 60,000 instances, and the Batchsize we choose is fixed at 128. Then every round of training is performed, the number of All-reduce operations required is fixed, as:

3 $$\begin{aligned} K=60000/128 \end{aligned}$$

Although the number of processes involved in each iteration is different, the size of the data block that a single process interacts with is the size of the weight W of the constant neural network, which is counted as W,

4 $$\begin{aligned} S=W \end{aligned}$$

Therefore, formula (1) can be rewritten as:

5 $$\begin{aligned} T2={458.75\times \log }_2N{\left( \alpha +{W}/{B}+W\times C\right) } \end{aligned}$$

Therefore, it can be seen from the above formula that when the communication bandwidth and the calculation cost per bit are determined, the overall communication time is only related to the number of processes and the communication delay between the two processes. Besides, when the number of processes is odd, the communication overhead of each batch will increase by 1 unit time, so the total communication overhead time when the number of processes is odd is:

6 $$\begin{aligned} T2=468.75\times \left[ \log _2{N\left( \alpha +{W}/{B}+W\times C\right) }+1\right] \end{aligned}$$

Therefore, with the exception of the FTP single-node experiment, in all other test scenarios, the total communication time increases with the increase of the number of processes, because the change in communication delay increases with the number of processes when the number of processes is small. There is little change, so the total communication time *T*2 in the above formula is mainly affected by the number of processes N. *T*2 increases with the increase of N, but as N increases, the magnitude of the increase of *T*2 decreases. Consistent with the trend of the logarithmic curve. Further, this article found that in the training scenario of FTP single node, when the number of processes exceeds 20, the overall communication time will decrease as the number of processes increases, because when the number of processes is relatively large, the communication delay $\alpha$ and the communication bandwidth B between processes will become complicated and cannot be generalized. Therefore, the calculation method of the above communication delay should be described as:

7 $$\begin{aligned} T2=468.7\times \left[ \sum _{i=1}^{N}{\log _2{\left( \alpha _i+{W}/{B_i}+W\times C\right) }+1}\right] \end{aligned}$$

It is found from the experiment that according to the above formula, when the number of processes continues to increase, the overall communication delay between the processes decreases and the overall communication bandwidth increases, resulting in a decrease in the total communication time.

## Conclusion

We evaluate the distributed training performance of the Tianhe-3 prototype for deep neural networks using the improved LeNet model, the classical AlexNet, VGG16, and ResNet18 models under our own ported pytorch distributed framework and propose a dynamic selection algorithm for the underlying communication mechanism. Our results can be used to guide the software and hardware design of Tianhe-3 as it moves toward E-class computing. For a comprehensive evaluation and illustration of the results, we design comprehensive and detailed experiments for single-node and multi-node clusters of FTP and MTP, respectively, providing software developers and hardware architects with multiple perspectives for future performance optimization.

In addition, we compare the performance of FTP and MTP processors, which shows the advantages and disadvantages of different processor architecture designs. It is hoped that we will provide a reference to the HPC community and Tianhe-3 developers to benefit the Chinese E-class supercomputer program, thus opening the way for the pursuit of E-class supercomputer development. In future work, we hope to combine the hardware features of the Tianhe-3 prototype compute nodes and the characteristics of the network topology to perform finer-grained tuning of the existing neural network distributed training framework structure on more platforms besides Pytorch to better exploit the potential computational power of the Tianhe-3 prototype.
